# Identifying Demographic Trends in the Use of Audio-Video and Audio-Only Telehealth by Arizona Medicaid Beneficiaries Before and During the COVID-19 Pandemic

**DOI:** 10.1007/s11606-024-09196-6

**Published:** 2024-11-25

**Authors:** Shreyas Hallur, Sara Salek, Sohail Daulat, Pamela Garcia-Filion

**Affiliations:** 1 Arizona Health Care Cost Containment System (AHCCCS), Phoenix, AZ USA; 2 Duke-Margolis Center for Health Policy, Duke University, Durham, NC USA; 3 Green Templeton College, University of Oxford, Oxford, UK; 4 Department of Biomedical Informatics, University of Arizona College of Medicine – Phoenix, Phoenix, AZ USA; 5 Department of Physiology, University of Arizona College of Medicine – Tucson, Tucson, AZ USA

**Keywords:** health equity, Medicaid, telehealth, COVID-19

## Abstract

**Introduction:**

For the first time, our study tracked disparities in the utilization of audio-video and audio-only telehealth for outpatient visits before and during the COVID-19 pandemic.

**Methods:**

Using a dataset of de-identified claims corresponding to telehealth and in-person visits, a retrospective cohort study was conducted for all beneficiaries continuously enrolled in Arizona Medicaid between October 2019 and November 2020. Our definition of telehealth only covered outpatient services delivered remotely via the audio-video or audio-only modality. Outcomes of interest were indicators of telehealth (vs. in-person) service delivery and audio-video (vs. audio-only) delivery of a telehealth service. Multivariate models evaluated the association between each outcome and demographic factors, including age, urban/rural location, sex, and race/ethnicity.

**Results:**

In this cohort study of 1,799,537 beneficiaries, age over 75, male sex, Asian race, Black race, Hispanic ethnicity, and Native American race were associated with reduced odds of telehealth use for outpatient visits pre-pandemic. These deficits persisted for all groups except the Black race after the pandemic’s onset. Throughout the study period, older age and Native American race were correlated with greater audio-video use while Black race indicated reduced odds of audio-video use. Hispanic ethnicity indicated lower odds of audio-video use only during the pandemic. Rural members exhibited greater odds of both overall telehealth and audio-video use for outpatient visits prior to the pandemic but both trends reversed as a rural-urban divide emerged during the pandemic. Spearman correlations between broadband access and audio-video uptake yielded no significant results pre-pandemic but a strong correlation emerged during the pandemic.

**Discussion:**

Pandemic-era telehealth expansions reduced but did not eliminate pre-existing disparities in telehealth and audio-video utilization for outpatient visits, indicating a need for health systems to better engage minority, elderly, and rural populations and continue to support audio-only telehealth.

## INTRODUCTION

The coronavirus disease 2019 (COVID-19) pandemic sparked an unprecedented expansion of telehealth coverage. Many insurers authorized virtual delivery of medical care for the first time while implementing payment parity for services offered via telehealth. These policies led to record utilization of virtual care for outpatient visits relative to pre-pandemic levels, such as a 63-fold increase in telehealth among Medicare beneficiaries^1^ and a 56-fold increase among Anthem Blue Cross Blue Shield members.^2^ Arizona’s Medicaid agency, the Arizona Health Care Cost Containment System (AHCCCS), saw a smaller, but significant 839% increase in telehealth claims.

Despite pandemic-era telehealth expansions, many studies have identified disparities in telehealth utilization across demographic groups.^3–8^ Reduced access to computers and broadband internet among many older,^9^ rural,^10^ minority,^11^ and low-income individuals^12^ may have limited their access to live video telehealth services. Access to audio-only modalities that rely only on telephones could help alleviate these concerns, especially for follow-up appointments^13^ or chronic disease management,^14^ but overreliance on this technology could introduce quality concerns in cases where nonverbal communication is important to delivering medical care.^15^ Faced with this uncertainty, Arizona specifically adopted the place of service code “02” to track the delivery of an outpatient service via audio-only means. Although this modifier was retired in 2021, the place of service “02” indicator uniquely allowed AHCCCS to track audio-only utilization at a time when national coding systems lacked such a metric.

These indicators have empowered AHCCCS to adjust its policies on audio-only telehealth as necessary to balance member health and service accessibility. In October 2019, AHCCCS reformed its coverage of outpatient services delivered via telehealth by removing geographic limitations and allowing reimbursement of services administered from the member’s home.^16^ AHCCCS later expanded telehealth reimbursement during the pandemic to include a broader array of services.^17^ In May 2021, HB 2454 clarified Arizona Medicaid’s policy on audio-only telehealth, authorizing its use only when a member’s preferences, health, or technological barriers limited audio-video alternatives.^18^ The agency also set up Arizona Telehealth Subcommittees to continue monitoring audio-only telehealth. In December 2021, these committees recommended a reduction of audio-only codes for health insurers in Arizona, but reversed this decision in February 2022 to align Arizona Medicaid more closely with Medicare guidelines.^19^ And when the pandemic public health emergency ended in May 2023, AHCCCS disallowed the use of the audio-only modality for Evaluation and Management services, in line with CPT guidelines.^20^

Although monitoring audio-only utilization over time greatly benefited Arizona Medicaid’s decision-making, few studies have examined this trend before and during the pandemic. By analyzing Medicaid claims and encounter data from Arizona across three 4-month intervals, this study aims to describe which groups of beneficiaries were most likely to (1) utilize a telehealth service for outpatient visits relative to an in-person appointment and (2) use audio-video services (vs. audio-only services).

## METHODS

### Study Design

We conducted a retrospective cohort study of Medicaid beneficiaries to examine patterns in the utilization of telehealth and in-person care during the pandemic. To ensure a fair comparison with in-person visits, telehealth in this study refers to an authorized outpatient audio-only or audio-video visit with a medical provider. We extracted de-identified demographic and claims data for all beneficiaries enrolled in Arizona Medicaid. This study only included members continuously enrolled between October 1, 2019 and November 30, 2020, to ensure cohort composition did not influence any trends in the utilization of telehealth and in-person services. Collected demographic information included self-identified race and ethnicity, age on the date of service, urban/rural status (as determined by Rural–Urban Community Area Codes^21^), and sex. This analysis followed the Strengthening the Reporting of Observational Studies in Epidemiology (STROBE) reporting guidelines.

The study period included three 4-month intervals. The first window (pre-pandemic) spanned from October 1, 2019, the day Arizona Medicaid initially expanded its telehealth codeset, to January 31, 2020, a cutoff point chosen to match the 4-month duration of the other two intervals. The next interval (first wave) covered April 1 to July 31, 2020, a 4-month period that roughly captures the first wave of COVID-19 cases in Arizona. Utilization data for March was skipped to avoid complications in telehealth coding during Arizona’s transition into lockdown. The last window (fall 2020) ranged from August 1 to November 30, 2020, a period of relative decline in the number of COVID-19 cases. In each interval, we tracked the utilization of in-person and remotely delivered outpatient services across the full study population.

For each 4-month period, the primary outcomes of interest were (1) whether a recorded outpatient visit corresponded to an in-person or telehealth service and (2) whether a submitted telehealth claim corresponded to an audio-video service (as opposed to an audio-only service). An audio-video claim was identified by the “GT” modifier while the use of the “UD” modifier or place of service code “02” indicated audio-only delivery. We excluded asynchronous telehealth claims, which include remote patient monitoring, e-consults, and store-and-forward services, from our dataset.

### Data Analysis

Self-identified race and ethnicity information were united into a singular race/ethnicity variable in accordance with prior literature.^3,22,23^ All individuals of Hispanic ethnicity were coded as Hispanic, and all other race/ethnicity groups only included non-Hispanic members. Our study also excluded claims from Indian Health Service facilities and tribally operated 638 health programs since they were not required to submit telehealth modifiers. These institutions account for about half of all claims for Native American members, so our sample does not represent all telehealth or in-person utilization by this population.

For each demographic group and time interval, we aggregated the number of telehealth users and telehealth claims per 1000 members. We also computed the proportion of telehealth users and claims attributable to a demographic group by dividing the counts of each demographic group by total figures across all groups for a given 4-month interval. Chi-square tests were used to assess demographic differences between the population that did and did not use at least one telehealth service as well as the number of audio-video and audio-only claims used by each demographic group. Adjusted odds ratios for telehealth and audio-video utilization by demographic group were calculated at the visit level via logistic regression models where the presence of a telehealth indicator or an audio-video indicator, respectively, served as the dependent variable. Claims for in-person visits were excluded from the regression model comparing use of the audio-video and audio-only telehealth modalities. Independent variables for these regressions included age, location, sex, and race/ethnicity.

We also assessed the Spearman correlation between audio-video use and broadband access by location for the full study population before and during the pandemic. For this analysis, we used the 2019 5-year American Community Survey estimate of broadband access by census Public Use Microdata Area for Arizonans enrolled in Medicaid.^24^ All statistical analyses were conducted in R version 3.6.3,^25^ with a two-tailed *p* value less than 0.05 selected as the threshold for statistical significance.

## RESULTS

A total of 1,799,537 members were continuously enrolled in Arizona Medicaid between October 1, 2019 and November 30, 2020. In total, 115,270 distinct members used a telehealth service during the pre-pandemic period, but this number surged to 404,057 and 365,775 beneficiaries during the first wave and fall 2020 intervals, respectively. Table 1 displays telehealth users by demographic group. Across all three time periods, younger (0–17), older (65 +), rural, Asian, Black, and Native American members were underrepresented among telehealth users. The gaps in telehealth utilization narrowed for seniors and racial minority groups during the pandemic but widened for children and rural members.

**Table 1:** Demographics of AHCCCS Population and the Number (Proportion) of Members Who at Least Have One Telehealth Service Across 3 Time Periods, Subdivided by Demographic Group

Characteristic	Overall AHCCCS population		Number of telehealth users					
			Before the pandemic (October 2019–January 2020)		During first wave (April–July 2020)		In fall 2020 (August–November 2020)	
Age								
0–17	710,517	(39.48)	40,918	(35.50)	125,236	(30.99)	116,534	(31.86)
18–49	706,688	(39.27)	49,343	(42.81)	169,235	(41.88)	154,132	(42.14)
50–64	229,494	(12.75)	20,360	(17.66)	79,879	(19.77)	69,891	(19.11)
65–74	94,286	(5.24)	3971	(3.44)	19,512	(4.83)	16,779	(4.59)
75 +	58,552	(3.25)	678	(0.59)	10,195	(2.52)	8439	(2.31)
Location								
Rural	264,210	(14.68)	14,522	(12.60)	41,656	(10.31)	35,587	(9.73)
Urban	1,535,327	(85.32)	100,748	(87.40)	362,401	(89.69)	330,188	(90.27)
Sex								
Female	975,309	(54.20)	59,266	(51.41)	230,712	(57.10)	207,487	(56.73)
Male	824,228	(45.80)	56,004	(48.59)	173,345	(42.90)	158,288	(43.27)
Race/ethnicity								
Asian	34,795	(1.93)	886	(0.77)	6037	(1.49)	5232	(1.43)
Black	121,021	(6.73)	7164	(6.21)	26,815	(6.64)	24,544	(6.71)
Hispanic	676,895	(37.61)	28,161	(24.43)	129,863	(32.14)	114,722	(31.36)
Native American	146,891	(8.16)	3253*	(2.82)	16,200*	(4.01)	14,992*	(4.10)
White	482,803	(26.83)	45,783	(39.72)	136,372	(33.75)	123,866	(33.86)
Unknown	337,132	(18.73)	30,023	(26.05)	88,770	(21.97)	82,419	(22.53)
Total	1,799,537		115,270		404,057		365,775	

Chi-square analyses yielded *p* < .05 for all demographic groups for all three time intervals

^*^Telehealth use figures based off partial claims data

Table 2 depicts the baseline differences in the number of audio-video and audio-only claims submitted by each demographic group alongside the proportion of total claims attributable to each group. As expected, telehealth utilization surged during the first wave period relative to pre-pandemic levels, with an 18- and fourfold increase in the number of audio-video and audio-only claims, respectively. Although the volume of audio-only claims declined by 14.4% between the first wave and fall of 2020, the number of audio-video claims increased by 4.1%. Throughout the study interval, the number of audio-only claims generally exceeded the count of audio-video claims. However, children, seniors (65 +), and Native American members all exhibited greater audio-video use during the fall 2020 period.

**Table 2:** Number (Proportion) of Claims Submitted for Audio-Video and Audio-Only Telehealth Services per 1000 Members Across 3 Time Periods, Subdivided by Demographic Group

Characteristic	Claims per 1000 members											
	Before the pandemic (October 2019–January 2020)				During the first wave (April–July 2020)				After the first wave (August–November 2020)			
	Audio-video		Audio-only		Audio-video		Audio-only		Audio-video		Audio-only	
Age												
0–17	15.92	(21.91)	216.16	(35.34)	643.51	(47.60)	788.07	(31.68)	715.59	(50.83)	650.89	(30.59)
18–49	39.94	(54.68)	252.98	(41.14)	469.79	(34.57)	1099.72	(43.98)	482.87	(34.11)	963.82	(45.05)
50–64	44.98	(20.00)	367.70	(19.42)	543.58	(12.99)	1568.84	(20.37)	488.62	(11.21)	1346.27	(20.43)
65–74	14.54	(2.66)	169.45	(3.68)	319.52	(3.14)	630.68	(3.36)	269.23	(2.54)	537.87	(3.35)
75 +	6.61	(0.75)	31.02	(0.42)	279.51	(1.70)	182.15	(0.60)	224.11	(1.31)	148.42	(0.57)
Location												
Rural	40.98	(20.98)	170.36	(10.36)	255.83	(7.04)	686.53	(10.26)	230.54	(6.09)	552.31	(9.65)
Urban	26.56	(79.02)	253.71	(89.64)	581.55	(92.96)	1032.93	(89.74)	611.86	(93.91)	889.73	(90.35)
Sex												
Female	26.03	(49.20)	234.38	(52.61)	509.68	(51.76)	988.18	(54.53)	517.28	(50.43)	852.11	(54.97)
Male	31.81	(50.80)	249.87	(47.39)	562.17	(48.24)	974.84	(45.47)	601.54	(49.57)	826.09	(45.03)
Race/ethnicity												
Asian	10.92	(0.74)	92.92	(0.74)	282.77	(1.02)	478.92	(0.94)	298.92	(1.04)	396.29	(0.91)
Black	22.45	(5.26)	239.10	(6.66)	463.19	(5.84)	974.72	(6.67)	489.01	(5.92)	851.35	(6.81)
Hispanic	19.08	(25.03)	140.98	(21.96)	330.23	(23.27)	636.80	(24.39)	357.39	(24.18)	541.05	(24.22)
Native American	13.54	(3.85)	68.51	(2.32)	332.42	(5.08)	418.51	(3.48)	367.90	(5.40)	323.42	(3.14)
White	66.71	(43.58)	505.48	(39.22)	1037.25	(36.41)	2005.28	(38.25)	1019.07	(34.35)	1708.59	(38.10)
Unknown	23.03	(21.54)	261.95	(29.10)	564.45	(28.37)	961.22	(26.26)	603.18	(29.11)	839.62	(26.81)
Average	28.68		241.47		533.73		982.07		555.87		840.19	

Chi-square analyses yielded *p* < .05 for all demographic groups for all three time intervals

**Figure 1:**
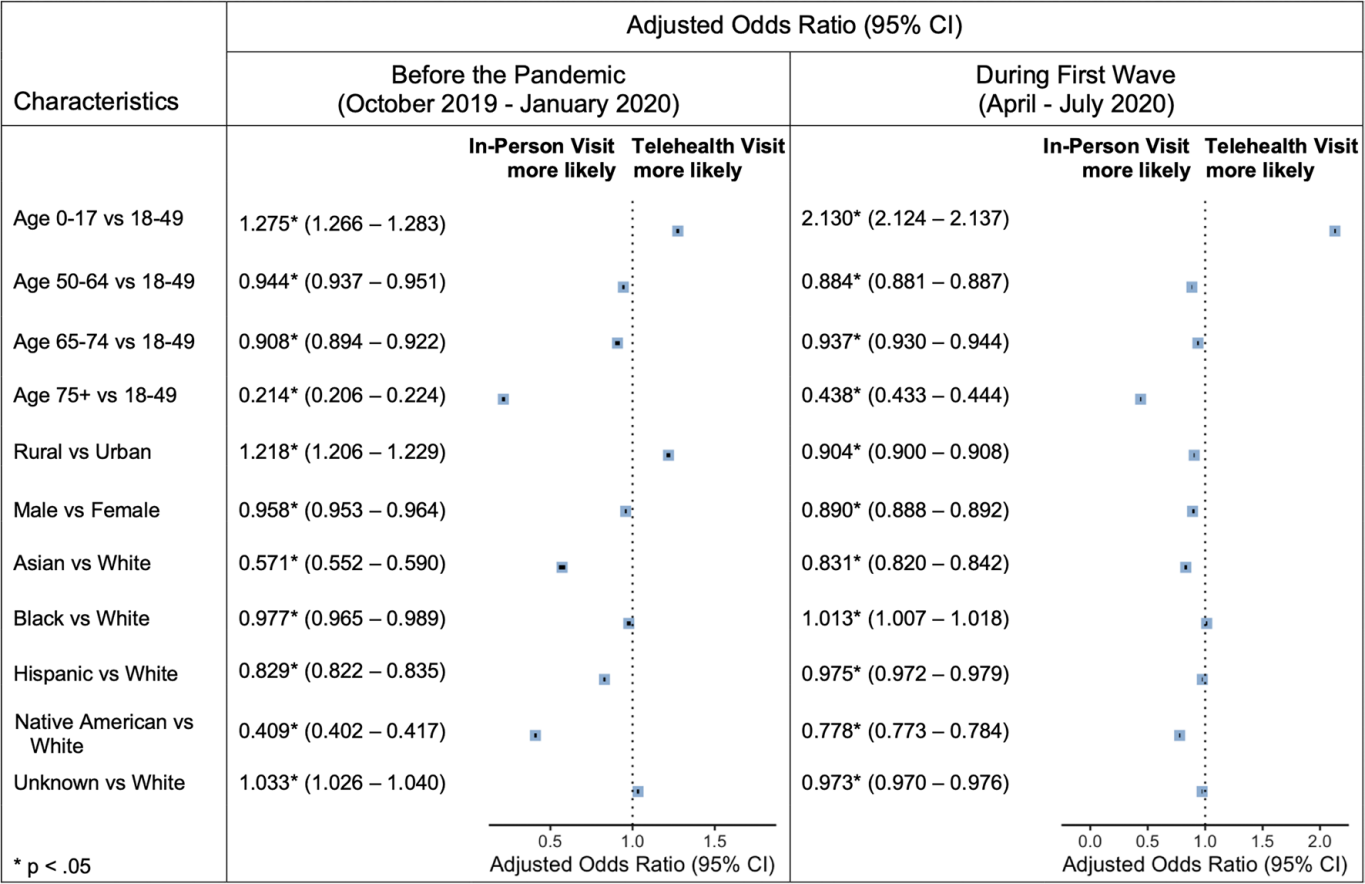
Forest plots depicting adjusted odds ratios for any telehealth use (relative to in-person use) among AHCCCS members before the pandemic and during the first wave.

Figure 1 displays the patient demographics associated with telehealth utilization for the pre-pandemic and first-wave intervals. Telehealth use surged for all groups during the pandemic, so any observed changes over time represent a difference in the relative utilization rates between groups. Seniors initially exhibited much greater use of in-person care when compared to adults aged 18–49 (adjusted odds ratio [aOR] 0.214 [95% confidence interval 0.206–0.224]) while children displayed greater use of telehealth services (aOR 1.275 [95% CI 1.266–1.283]). Both trends continued into the pandemic. Conversely, rural location was first associated with greater telehealth adoption (aOR 1.218 [95% CI 1.206–1.229]), but this trend reversed during the pandemic (aOR 0.904 [95% CI 0.900–0.908]). The male sex, Asian race, Hispanic ethnicity, and Native American race were all associated with lower telehealth adoption rates before and during the pandemic relative to the female sex and White race, respectively. While the gap between sexes increased during the first wave, the observed racial and ethnic gaps in telehealth adoption slightly narrowed in the same period. By contrast, utilization patterns for Black members closely resembled those of White members before (aOR 0.977 [95% CI 0.965–0.989]) and during the pandemic (aOR 1.013 [95% CI 1.007–1.018]).

**Figure 2:**
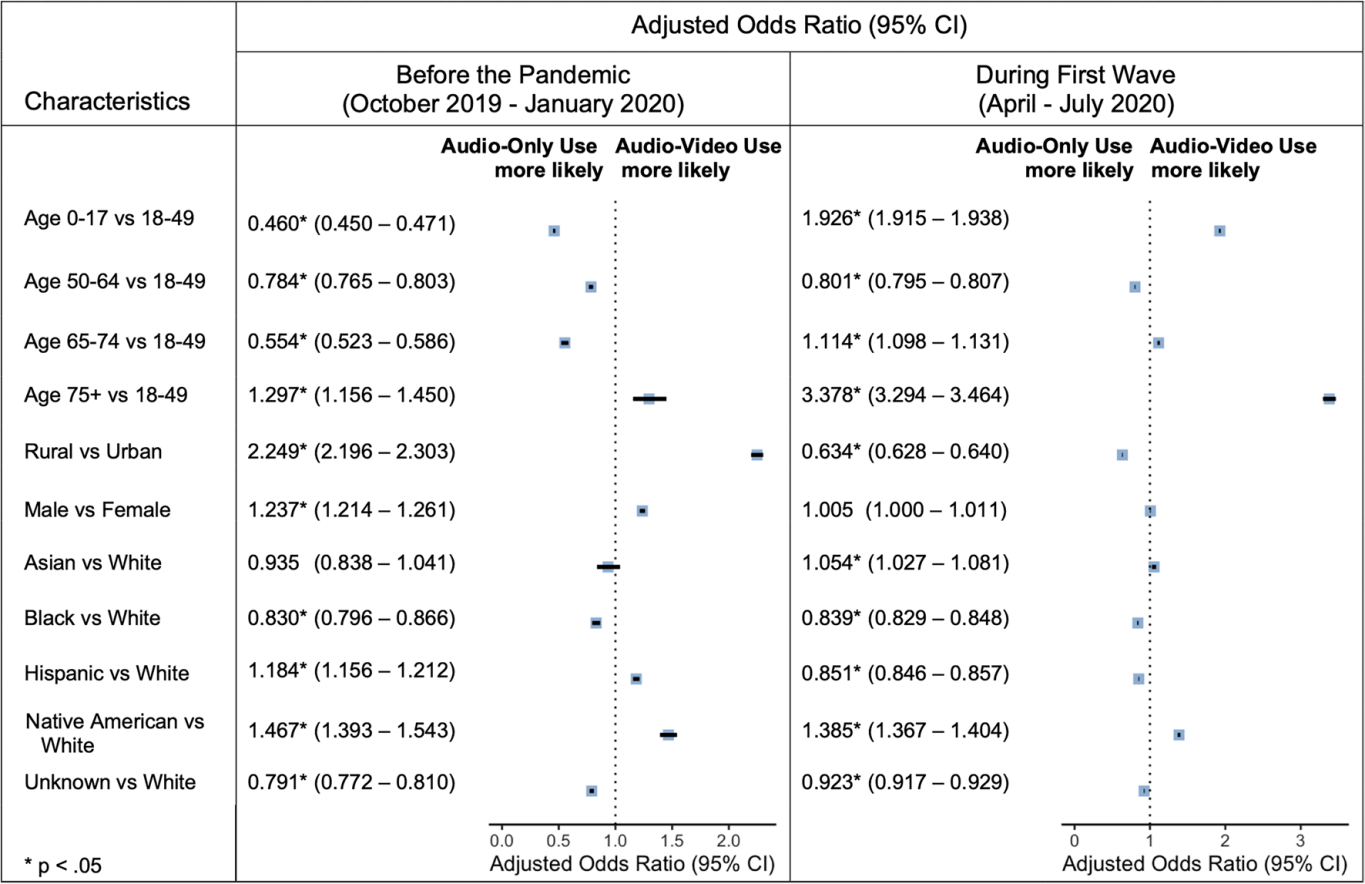
Forest plots depicting adjusted odds ratios for audio-video claims (relative to audio-only claims) by AHCCCS members before the pandemic and during the first wave.

When considering all submitted telehealth claims, Fig. 2 depicts the patient characteristics associated with audio-video visits on multivariate analysis. Although children’s adoption of audio-video visits was limited before the pandemic relative to adults aged 18–49 (aOR 0.460 [95% CI 0.450–0.471]), their uptake increased significantly during the first wave (aOR 1.926 [95% CI 1.915–1.938]). Conversely, older seniors (75 +) exhibited a tendency to access audio-video services relative to adults aged 18–49 that only increased during the pandemic. Rural location was initially associated with audio-video use (aOR 2.249 [95% CI 2.196–2.303]), but this trend reversed between April and July 2020 (aOR 0.634 [95% CI 0.628–0.640]). While male sex was strongly associated with audio-video use between October 2019 and January 2020 (aOR 1.237 [95% CI 1.214–1.261]), this gap did not manifest during the pandemic. Relative to the White race, the Black race was associated with less audio-video use both before and after the pandemic’s onset. There was no statistical difference in audio-video use between members of the Asian race and the White race before the pandemic, but the Asian race was associated with slightly greater odds of audio-video use during the first wave (aOR 1.054 [95% CI 1.027–1.081]). The opposite trend emerged for the Hispanic ethnicity, with it being linked to more audio-video use relative to the White race between October 2019 and January 2020 (aOR 1.184 [95% CI 1.393–1.543]) and less audio-video use during the first wave (aOR 0.851 [95% CI 0.846–0.857]). Native Americans were the only minority group to exhibit a strong association with audio-video use across all time points.

**Figure 3:**
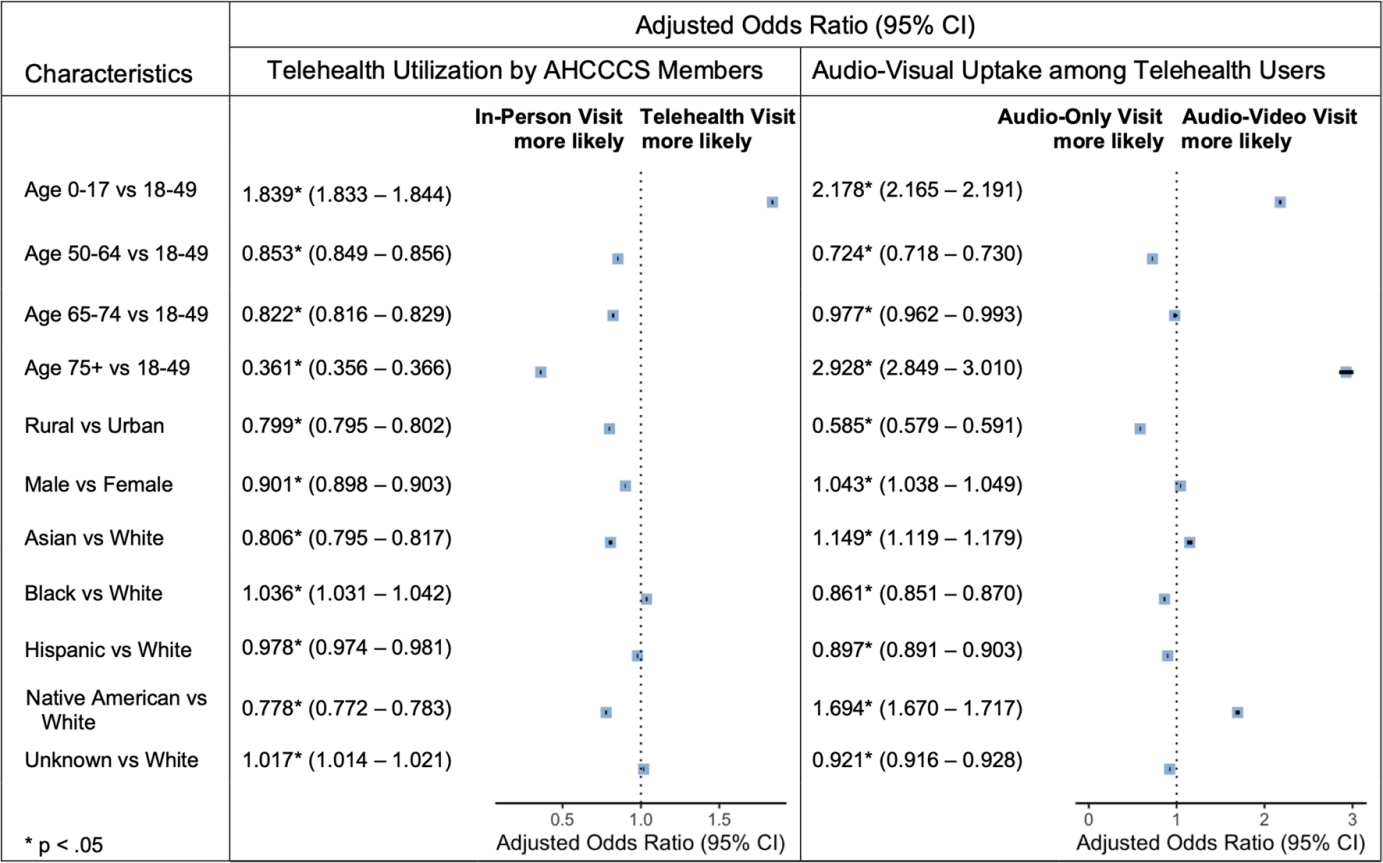
Forest plots depicting adjusted odds ratios for telehealth and audio-video utilization by demographic group between August and November 2020.

Adjusted odds ratios were also calculated for the August to November period (Fig. 3), but there were no major differences in utilization trends relative to the April to July period.

**Figure 4:**
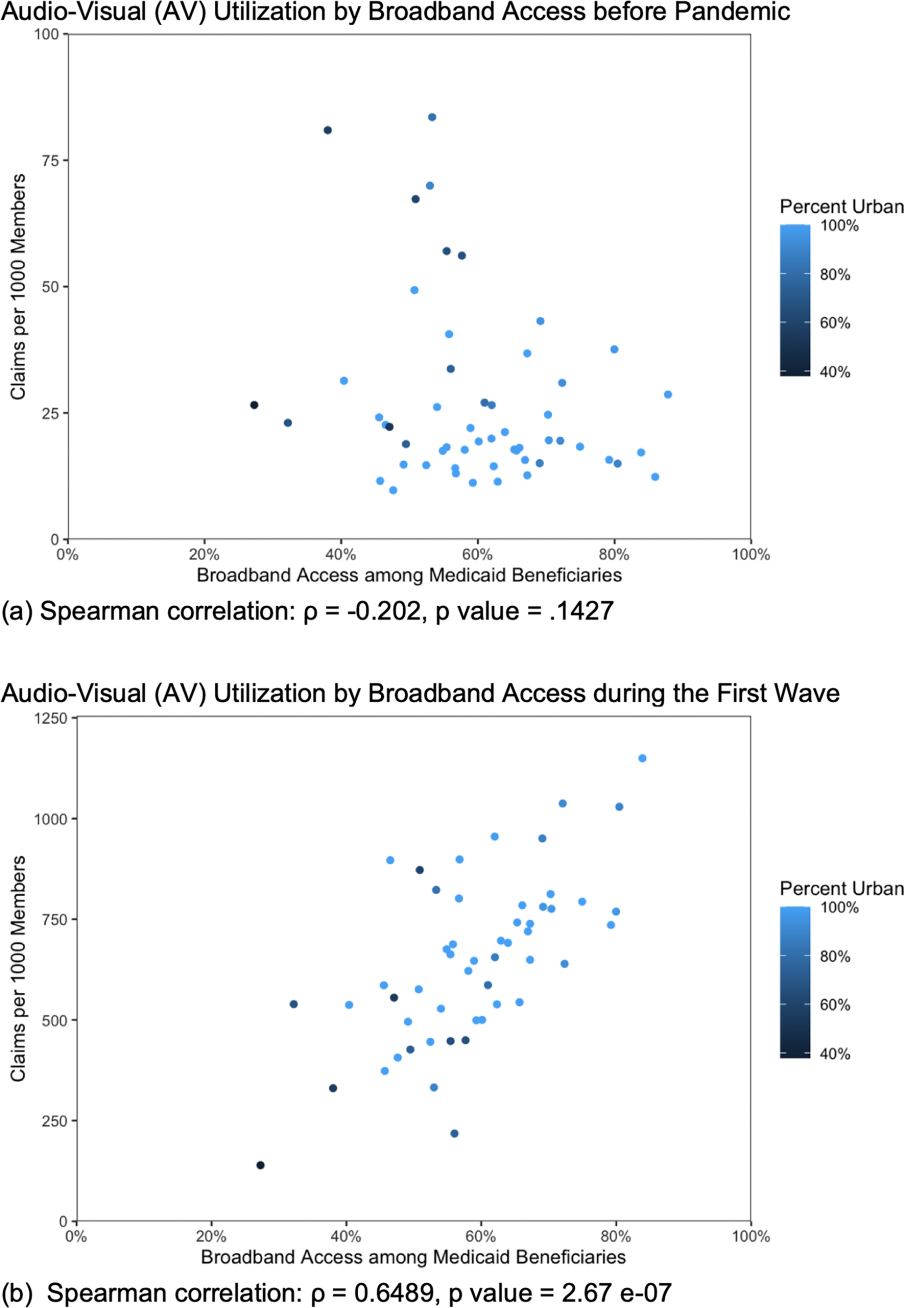
Spearman correlations between per-member utilization of audio-video telehealth and access to broadband internet by census Public Use Microdata Area.

The Spearman correlation tests of audio-video utilization by broadband access and location yielded a statistically insignificant association pre-pandemic and a positive association during the pandemic periods (pre-pandemic: Spearman’s *ρ* = − 0.202 [*p* = 0.143], first wave: Spearman’s *ρ* = 0.649 [*p* < 0.05], fall 2020: Spearman’s *ρ* = 0.639 [*p* < 0.05]). Figure 4 visualizes the correlation between broadband access and audio-video utilization for each census Public Use Microdata Area in Arizona for the fall 2020 and first wave intervals.

## DISCUSSION

We present the first study, to our knowledge, that examines trends in telehealth uptake by demographic group and modality both before and after the pandemic’s onset.

Relative to in-person visits, the telehealth utilization trends identified in this study largely reflect patterns that appear in prior literature. We found that seniors exhibited lower use of telehealth services across all time periods, but this gap narrowed during the pandemic. These findings align with previous studies^3,4,6,26–29^ and likely reflect seniors’ reduced rates of device ownership and technological literacy when compared to working-age adults.^30^ Conversely, women exhibited greater telehealth use across all time points, consistent with previous studies.^3,4,26–29^ We identified consistently lower odds of telehealth use among Asian^3,8^ and Hispanic^8^ populations across all time points of interest. These observed gaps narrowed during the pandemic in both our study and other longitudinal inquiries.^28,29^ Although the Black race was associated with lower odds of telehealth use pre-pandemic relative to the White race, Black members exhibited greater odds of telehealth use after March 2020, contradicting other sources that found enduring divides between these two populations.^4,27,31^ This period of telehealth adoption coincided with the emergence of a new urban–rural divide. Rural Medicaid beneficiaries exhibited relatively lower odds of telehealth uptake relative to their urban counterparts only during the pandemic, which aligns with previous literature.^6,28,29,32^ Future work should identify the outcomes associated with these telehealth patterns, in line with prior research conducted with the Veterans Health Administration.^33^

By contrast, our analysis of audio-video and audio-only telehealth adoption highlights a number of novel insights. Seniors in our inquiry exhibited greater odds of audio-video use relative to working-age adults during the pandemic, but past studies have found the opposite trend.^3,4,7,34,35^ This discrepancy may stem from Medicaid’s role as the primary funder of long-term care, where support staff can help elderly members connect to audio-video telehealth. Furthermore, our analysis identified lower audio-video uptake among women only in the pre-pandemic period, but other sources have identified this bias during the pandemic as well.^3^ Our divergence from prior studies may stem from Arizona Medicaid’s unique role in insuring pregnant patients. When the pandemic introduced barriers to in-person care, pregnant women’s preference for audio-video telehealth could have contributed to the parity we observed across male and female beneficiaries.^36^ Our regression found no statistical difference in the odds of audio-video uptake among Asian and White members before the pandemic. While no studies date back to this period, our analysis of audio-video trends after the pandemic’s onset aligned with prior literature in finding that Asian members exhibited greater uptake of audio-video use relative to White members.^7^ In the pre-pandemic period, Black members had lower odds of video use than their White counterparts while Hispanic beneficiaries exhibited the opposite trend. However, Black and Hispanic enrollees both exhibited lower odds of audio-video use relative to their White peers during the pandemic, a trend corroborated by available literature.^3,4,7,34^ Our longitudinal analysis suggests that telehealth expansions during the pandemic have largely decreased inequities in audio-video use, although disparities still exist for Black and Hispanic members.

Finally, our regression of audio-video uptake by location reflects an emerging urban–rural divide. In the pre-pandemic period, rural members displayed significantly greater odds of video use relative to their urban counterparts, aligning with trends from previous research.^37^ However, the onset of the pandemic sparked a surge in urban audio-video utilization as members across the state sought alternatives to in-person care. Since rural audio-video use did not rise at the same rate, urban members now exhibited greater odds of video use compared to their rural peers. A moderately strong positive correlation between audio-video use and broadband access emerged only during the pandemic while no statistically significant correlation existed in the pre-pandemic interval. Given the sudden appearance of this trend, it is possible that limited broadband access restricted the uptake of audio-video telehealth in rural areas during the pandemic.

Our findings have corroborated disparities identified in the literature and found lower odds of telehealth uptake by rural, elderly, Asian, and Hispanic members during the pandemic. Telehealth expansion has moderated many of these divides relative to the pre-pandemic window, but their persistence nonetheless highlights the need for a policy response. Our longitudinal analysis of telehealth delivery highlights the need to continue supporting audio-only telehealth, which was the modality disproportionately accessed by rural, Black and Hispanic beneficiaries during the pandemic. Although some Medicaid agencies, including Arizona, have maintained audio-only flexibilities, at least four states have discontinued Medicaid reimbursement for audio-only services.^38^ Likewise, Medicare plans to discontinue the delivery of outpatient services via the audio-only modality for all service lines except behavioral and mental health by December 31, 2024.^39^ Given the reduced odds of audio-video use observed across all service lines in this study among Black, Hispanic, and rural Medicaid beneficiaries, these decisions could exacerbate existing inequities in telehealth access. National working groups on virtual care, such as those organized at the Veterans Health Administration,^40^ could help reconcile these differences while guiding the development of Medicaid telehealth policies at the state and national level.

### Limitations

Our investigation involves a few limitations. Given the observational nature of this study, we cannot extrapolate causal relations between demographic predictors and the observed disparities. Another constraint is that a significant proportion of our sample population identified as an Unknown race/ethnicity, potentially limiting the conclusions that can be drawn from our race and ethnicity analyses. Our analyses were also conducted at the visit-level, not the patient-level. Since Medicaid beneficiaries who completed more visits represent a larger part of our dataset, their utilization patterns may have biased our results. Finally, our decision to exclude claims from Indian Health Service facilities and tribally operated 638 health programs may have introduced sampling bias into the odds of telehealth and audio-video use we calculated for Native American members.

## CONCLUSIONS

In our analysis of de-identified Medicaid claims data, we found pandemic telehealth expansions have reduced, but not eliminated, disparities in telehealth and audio-video use across race, ethnicity, sex, and age. However, a new urban–rural divide in telehealth and audio-video uptake has emerged since the pandemic’s onset. Our work indicates a need to engage minority, elderly, and rural populations facing telehealth barriers while continuing to support the delivery of audio-only services.

## Data Availability

Raw data from our claims database are not publicly available to preserve the privacy of Arizona Medicaid beneficiaries under the Health Insurance Portability and Accountability Act of 1996.
